# IDEAL-D Framework for Device Innovation: A Consensus Statement on the Preclinical Stage

**DOI:** 10.1097/SLA.0000000000004907

**Published:** 2021-08-23

**Authors:** Hani J. Marcus, Amy Bennett, Aswin Chari, Toni Day, Allison Hirst, Archie Hughes-Hallett, Angelos Kolias, Richard M. Kwasnicki, Janet Martin, Maroeska Rovers, Sarah E. Squire, Peter McCulloch

**Affiliations:** ∗ Wellcome EPSRC Center for Interventional and Surgical Sciences, University College London, London, UK; † Department of Neurosurgery, National Hospital for Neurology and Neurosurgery, UCLH Foundation Trust, London, UK; ‡ Orthox Ltd., Oxford, UK; § Department of Neurosurgery, Great Ormond Street Hospital, London, UK; ¶ Institute of Child Health, University College London, London, UK; || OrganOx Ltd., Oxford, UK; ∗∗ Nuffield Department of Surgical Sciences, University of Oxford, John Radcliffe Hospital, Oxford, UK; †† Department of Urology, Charing Cross Hospital, Imperial College Healthcare NHS Trust, London, UK; ‡‡ Division of Neurosurgery, Department of Clinical Neurosciences, University of Cambridge, Cambridge, UK; §§ Surgery Theme, Cambridge Clinical Trials Unit, Cambridge University Hospitals, Cambridge, UK; ¶¶ Department of Surgery and Cancer, Imperial College London, UK; |||| Western University, Ontario, Canada; ∗∗∗ Departments of Health Evidence and Operating Rooms, Radboud University Medical Center, Nijmegen, The Netherlands; ††† Department of Physiology, Anatomy and Genetics, University of Oxford, Oxford, UK.

**Keywords:** devices, first-in-human, IDEAL, innovation, preclinical, regulation

## Abstract

**Objective:**

To extend the IDEAL framework for device innovation, IDEAL-D, to include the preclinical stage of development (stage 0).

**Background:**

In previous work, the IDEAL collaboration has proposed frameworks for new surgical techniques and complex therapeutic technologies, the central tenet being that development and evaluation can and should proceed together in an ordered and logical manner that balances innovation and safety.

**Methods:**

Following agreement at the IDEAL Collaboration Council, a multidisciplinary working group was formed comprising 12 representatives from healthcare, academia, industry, and a patient advocate. The group conducted a series of discussions following the principles used in the development of the original IDEAL framework. Importantly, IDEAL aims for maximal transparency, optimal validity in the evaluation of primary effects, and minimization of potential risk to patients or others. The proposals were subjected to further review and editing by members of the IDEAL Council before a final consensus version was adopted.

**Results:**

In considering which studies are required before a first-in-human study, we have: (1) classified devices according to what they do and the risks they carry, (2) classified studies according to what they show about the device, and (3) made recommendations based on the principle that the more invasive and high risk a device is, the greater proof required of their safety and effectiveness before progression to clinical studies (stage 1).

**Conclusions:**

The proposed recommendations for preclinical evaluation of medical devices represent a proportionate and pragmatic approach that balances the de-risking of first-in-human translational studies against the benefits of rapid translation of new devices into clinical practice.

New devices have preceded many of the major advances in clinical practice, especially in surgery. The process of translation of devices from the laboratory to clinical practice can be seen as analogous to the process of drug development, progressing through a predictable series of unique stages, each associated with their own challenges and risks. The context is, however, very different, because there are major differences in the nature of the developmental process, and these have resulted in markedly different regulatory environments. The result has been that rigorous scientific evaluation of devices, especially therapeutic devices, is generally acknowledged to lag behind that of new medicines. This difficulty has been recognized by the World Health Organization's 2014 resolution on Health Technology Assessment calling for “rigorous and structured research methodology and transparent and inclusive processes” for all types of health technology.^1^

An independent network of clinicians and scientists initially convened to provide a framework for evaluating innovation in surgical techniques, the idea, development, exploration, assessment, long-term follow-up (IDEAL) collaboration, has previously proposed a model for the clinical evaluation of device innovation, IDEAL-D.^2–6^ The central tenet of this framework is that development and evaluation can and should proceed together in an ordered and logical manner that balances innovation and safety (Fig. 1). Whilst for surgical operations procedural modification and optimization of new interventions largely takes place in the clinical environment, with devices this largely occurs before the first-in-human studies.^6^ Arguably the most challenging translational barrier for devices is taking their development to the point of a first-in-human study. As few as 1 in 10 devices developed in academia ever result in a first-in-human study.^7^ Any methodological framework for device evaluation that begins with the first clinical study, therefore, ignores the majority of the development process. For this reason, the IDEAL-D paper proposed an IDEAL stage 0 analogous to the phase 0 trials in the pharmaceutical industry, designed to efficiently assess whether a specific agent works as desired in humans before phase I testing.^8,9^ However that publication avoided making specific methodological recommendations for stage 0, for 2 main reasons. First, the potential range of studies that might be relevant is formidable, and second, in most jurisdictions a significant amount of evaluation work is prespecified by governmental regulatory bodies charged with ensuring the safety and effectiveness of devices, such as the Food and Drug Administration (FDA) in the United States and the national competent authorities in the European Union (EU).

**FIGURE 1:**
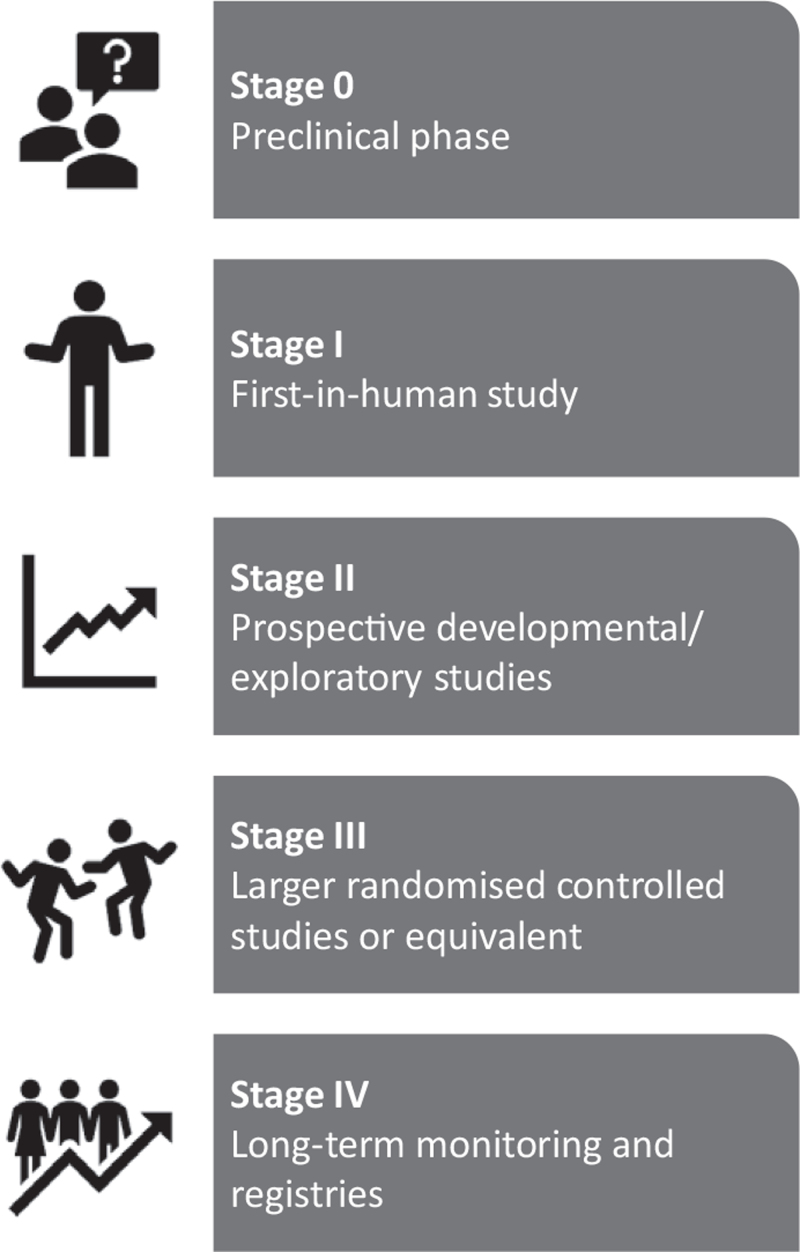
Schematic representation of the IDEAL-D (idea, development, exploration, assessment, long-term follow-up) stages of assessment for interventional therapy innovation, including the preclinical stage 0 being proposed here.

In this discussion paper a collaborative IDEAL group has developed initial outline proposals for rationalizing both the selection of studies and the methodology and reporting standards to be used in the preclinical stage of development (stage 0), applying the same ethical principles as were applied to clinical evaluation. This model has been developed in the context of existing regulatory structures, with the principal aim of providing a universally applicable, transparent, and robust framework for planning preclinical studies derived from ethical principles which can be applied across all healthcare settings.

## METHODS

Following agreement at the IDEAL Collaboration Council, a multidisciplinary working group was formed comprising 12 representatives from healthcare, academia, industry, and a patient advocate. Collectively the group included consultant surgeons, university professors, and industry experts with experience of device development and evaluation. The group conducted a series of discussions chaired by HJM, following the principles used in the development of the original IDEAL Framework. Importantly, IDEAL aims for maximal transparency, optimal validity in the evaluation of primary effects, and minimization of potential risk to patients or others. Where countervailing pressures limit our capacity to achieve these aims, this is highlighted and explained. The proposals were subjected to further review and editing by members of the IDEAL Council before a final consensus version was adopted.

We adopted a consensus-based approach to developing these recommendations. In considering which studies are required or desirable before a device is ready for a first-in-human study, we decided to divide the problem into 3 parts:

(1) What devices we are considering and how to stratify the level of risk in the devices?
(2) What types of studies might be needed?
(3) How we should decide on what to evaluate, and how rigorous should this evaluation be?

To help us answer these questions, we developed:

(1) A classification system based on what the devices do alongside a broadly applicable risk assessment system.
(2) A classification for study types based on what they show about the device.
(3) A risk-based approach to evaluation, taking the view that any predictable significant risk to patients requires evaluation sufficient to demonstrate conclusively that it can be managed or eliminated before human trials.

In developing our classification of devices we worked to ensure that our classification was founded on the IDEAL principles but compatible with both of the major regulatory device classification systems, rather than offering an alternative to them.

## RECOMMENDATIONS

### Classification of Devices

The most widely currently applied classifications for medical devices are those utilized by certifying bodies, such as the EU Council Directive (CE mark), and the FDA in the United States.

Broadly speaking, devices have been classified according to an ordinal scale of patient risk or by categorical factors (eg, associated specialty, intended use, duration of use, invasiveness, or surgical vs nonsurgical). The EU CE marking process utilizes a prescriptive set of 18 “rules,” associated with a series of algorithms to confirm class, and sub-class.^10^ The FDA process is less well defined with devices classified directly to 3 regulatory classes (I–III) based on the level of potential patient risk plus stratification amongst 16 medical specialty panels.^11^

We adopted a different approach to classification, designed to be consistent, easy to use, and to avoid duplication, but informed by both the FDA and EU classifications. Devices are classified according to a 3-tiered descriptive taxonomy, outlined in Table 1. The first 2 tiers classify the device as invasive or noninvasive, and surgical versus nonsurgical (see definitions below). The third tier is more descriptive and helps elucidate the potential harm a device may pose to a patient. This taxonomy leads to a classification compatible with the historic risk class system used by the FDA and CE marking processes, but which does not rely on the arbitrary decisions used in both systems. Although the table indicates the current EU device class, the simplicity and principles-based nature of this classification should ensure it can adapt to any changes in classification that may be introduced by either the EU or the FDA.

**TABLE 1:** Classification of Device Types. Software Used to Control Hardware is Classified According to the Associated Hardware. In Other Cases, Software is Classified as Noninvasive, and Either Nonsurgical or Surgical Depending on Their Use

		Device Classification Tiers		
Tier 1	Tier 2	Tier 3	Examples	Regulatory Class (EU)
Noninvasive	Nonsurgical	Noninvasive devices	Incision drapesWound dressings	Class I
			Tubing used with infusion pumpFridges for storing blood or tissue	Class IIa
			Dialysis systemsVentilators	Class IIb
			OrganOx metra perfusion circuit	Class III
	Surgical	Active therapeutic devices intended to administer or exchange energy	Lithotripsy devicesSurgical ultrasound devices	Class IIa or IIb
Invasive devices	Nonsurgical	Invasive with respect to body orifices	Indwelling urinary cathetersTracheal tubes	Class IIa
	Surgical	Surgical instruments	Suture needlesStaplers	Class IIa
			Cardiovascular cathetersExternal ventricular drains	Class III
		Absorbable surgical implants	Absorbable sutures	Class IIb
		Nonabsorbable surgical implants	Peripheral vascular grafts and stents	Class IIb
			Breast implantsTotal hip replacements	Class III

EU indicates European Union.

Consistent with existing conventions, any item that comes into contact or close proximity to any body cavity or open wound is defined as being invasive (tier 1).^12^ The distinction between surgical and nonsurgical is based on whether a device penetrates the surface of the body (tier 2). Invasive surgical devices are further sub-categorized according to whether the device is an instrument or an absorbable implant or a nonabsorbable implant (tier 3).

There are a number of important caveats to the IDEAL-D classification of devices. First, in line with current regulatory frameworks, software used to control a medical device falls under the classification of the associated hardware. Devices that replace epithelial surfaces (eg, skin or corneal allografts) are classed as implantable despite not breaching the body surface. Lastly, devices that use energy are only classified as active devices if they significantly convert the energy during their interaction with human tissues, for example, diathermy transforms electrical energy into heat to evaporate water in tissues and is, therefore, an active device, whereas surface electrodes for monitoring are not.

### Assessment of Risk

The device classification (Table 1) serves as a guide to the potential impact of the risk of a device, with the more invasive, surgical devices clearly carrying higher potential risks. However, we recognized that devices in the same category (eg, cardiovascular catheters and suture needles) may have differing risk profiles that warrant different evaluation strategies.

We, therefore, recommend a comprehensive, proactive analysis of the potential risks posed by a new device using an approach based on the principles of failure modes and effects analysis (FMEA) (Box 1). Investigators should systematically identify all foreseeable ways in which a device might fail and assess the likelihood and the probable impact of each possibility (Table 2A and B), and their product used to estimate the risk level using a matrix (Table 2C).

Box 1 **Failure Modes and Effects Analysis (FMEA)**

Systematically consider all possible failures

Analyze the possible effects of the failures

Determine the likelihood of these failures

Determine the severity of these failures

Multiply the likelihood by the severity to determine a risk level

Order the risk levels from highest-to-lowest

**TABLE 2:**
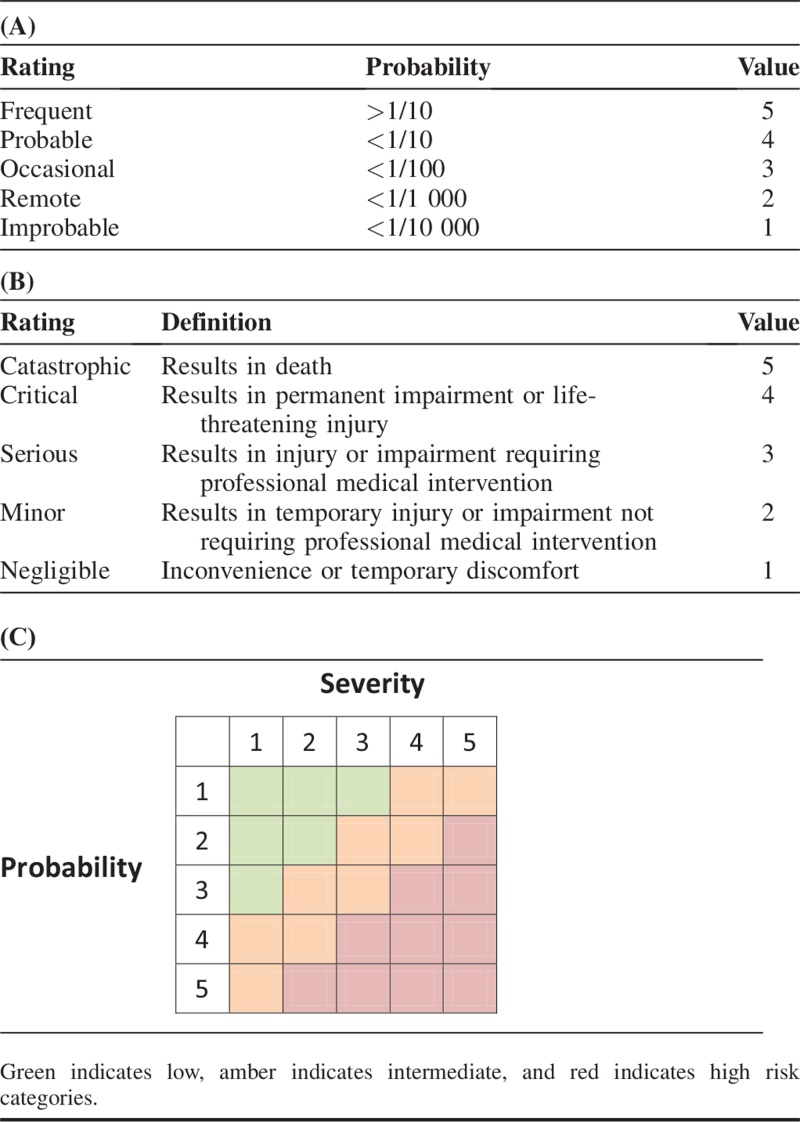
Failure Modes and Effects Analysis (FMEA) Approach to Stratifying Risk of Device Malfunction. Identification of the Likelihood (3A) and Severity (3B) of Device Failure Allows the Stratification of Risk (3C)

### Classification of Studies

**FIGURE 2:**
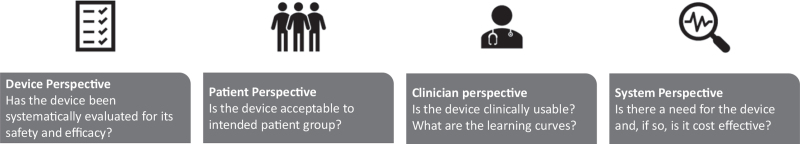
Schematic of classification of IDEAL-D stage 0 studies to incorporate the perspectives of all stakeholders. IDEAL-D indicates idea, development, exploration, assessment, long-term follow-up.

The range of different studies which might be appropriate before first use of a new device in humans is vast, and this represents the biggest challenge in developing recommendations for stage 0. We classified preclinical studies into 4 perspectives (system, patient, clinician, and device) to help investigators to consider all the aspects which might be important to evaluate. (Fig. 2). Evaluation for regulatory purposes has focused almost entirely on the device itself, and certain device studies are routinely required to comply with regulatory requirements. The aims and types of study that may be relevant to each category, including regulatory aspects, are outlined in Table 3 and explained below. All studies for regulatory purposes are expected to follow good laboratory practice, and we recommend that this quality standard should be observed for other evaluative studies also.

**TABLE 3:** Classification of Study Types

Perspective	Study Aim	Study Types	Regulatory Aspects
System	Device necessity	Unmet needs analyses	Not applicable
	Contextual relevance	Interaction analyses	Not applicable
	Economic viability	Economic modeling	Not applicable
Patient	Patient acceptability	Patient surveys	Risk management (ISO 14971); usability (human factors) (IEC 62366)
		Patient focus groups	Risk management (ISO 14971); usability (human factors) (IEC 62366)
Clinician	Clinical usability	Clinician surveys	Risk management (ISO 14971); usability (human factors) (IEC 62366); clinical investigation of medical devices for human subjects – good clinical practice (ISO 14155)
		Clinician focus groups	Risk management (ISO 14971); usability (human factors) (IEC 62366); clinical investigation of medical devices for human subjects – good clinical practice (ISO 14155)
Device	Device safety	Laboratory studies, for example, cytotoxicity	Risk management (ISO 14971): biological safety (ISO 10993); nonactive surgical implants (ISO 14603)
		Animal studies for toxicity, pyrogenicity	Risk management (ISO 14971): biological safety (ISO 10993); nonactive surgical implants (ISO 14603); medical electrical equipment (IEC 60601)
		Manufacturing simulations, sterility testing	Risk management (ISO 14971): sterilization of health care products (ISO 11737); aseptic processing (ISO 13408); packaging validation (ISO 11607); medical devices utilizing animal tissues and their derivatives (ISO 22442)
	Device effectiveness	Laboratory bench testing	Meets intended use (ISO 13485): nonactive surgical implants (ISO 14603); medical electrical equipment (IEC 60601)
		Laboratory simulations	Meets intended use (ISO 13485): nonactive Surgical implants (ISO 14603); medical electrical equipment (IEC 60601)
		Cadaver studies	Meets intended use (ISO 13485): nonactive surgical implants (ISO 14603); medical electrical equipment (IEC 60601)
		Animal studies	Meets intended use (ISO 13485): nonactive surgical implants (ISO 14603); medical electrical equipment (IEC 60601)

Note that International standards quoted within the table do not consider the harmonization processes required for conformity against the Medical Device Directive (undergoing transition at the time of print) for EU CE marking purposes. ICE indicates International Electrotechnical Commission; ISO, International Organization for Standardisation.

#### Device

Device perspective studies should evaluate both inherent safety by design and technical effectiveness. To avoid excessive complexity and cost, evaluation plans should focus on key aspects of the device that could affect safety or effectiveness. IDEAL-D stage 0 recommends using a risk-assessment based approach to identify characteristics of the device most likely to affect safety (eg, materials of construction, intended medical indication, whether the device is active or nonactive) and those essential for technical effectiveness (See Table 2).

Biological safety studies, including genetic, toxicological, and cytotoxicity assays and animal studies that review the local and systemic effects of implantable medical devices, and sterility testing, are specified and mandated by regulators in most jurisdictions. The practical relevance of these studies varies from case to case but because they are obligatory, IDEAL merely recommends that testing methods are adequate to establish safety in the manner expected.

#### Patient

Patient acceptability studies form part of the patient and public involvement aspect of research and the concept and definitions of “acceptability” continue to evolve.^13^ Testing the views of potential patients on need and acceptability should be considered, using focus groups before progression to stage 1.

#### Clinician

Studies from the clinician perspective capture clinician preferences, usability, and learning curves. Usability studies assess the effectiveness of the user interface (including any information for use) reducing the possibility of misuse. Design elements that incorporate and accommodate clinician preferences may improve device uptake. Studies of user learning curves should be performed to ensure adequate training is provided to clinicians before clinical use, preferably using realistic simulation, and may inform device design and progression to further stages.

#### System

System level studies seek to identify the gap in current healthcare that the new device would seek to fill. This includes establishing unmet needs using suitable surveys and focus groups, estimating potential health benefits and evaluating contextual relevance and health-economic implications using economic modeling, reviews of existing literature, and service evaluations.

### Risk Based Approach to Evaluation

**FIGURE 3:**
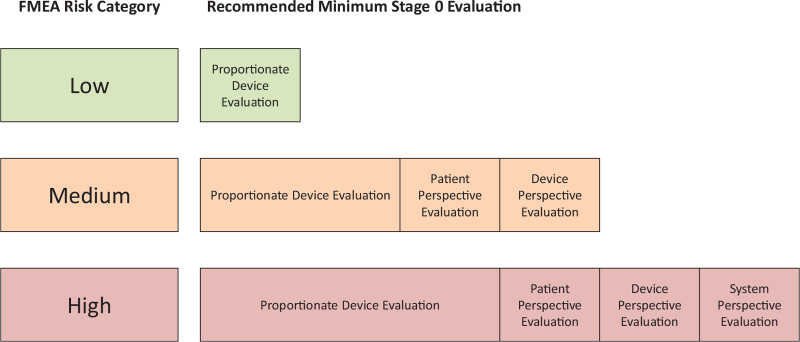
Graphical representation of the recommendation for stage 0 of the IDEAL-D framework, which balances the invasiveness and risk categories of the device (*x*-axis) against the thoroughness of the evaluation required to progress to stage I. IDEAL-D indicates idea, development, exploration, assessment, long-term follow-up.

An ethical approach to device evaluation should seek to provide a balance between the “goods” of facilitating timely innovation and of ensuring thorough safety evaluation, summarised in Figure 3.

All devices require device perspective studies. The rigor of the studies used to justify progression to use in patients should be calibrated against the potential risk to the patient, evaluated by the FMEA (Table 2). This will allow patients and clinicians to benefit quickly from low risk innovation whilst ensuring adequate safeguards for high risk devices. Significant risks of serious harm (amber or red in Table 2C) must be investigated thoroughly enough to identify the nature and likely effectiveness of possible countermeasures. Where the evidence required remains unclear, a group including representatives of innovators, clinicians, scientists, and patients could be asked to reach a consensus-based decision.

Patient and clinician perspective studies are recommended for devices with intermediate or high risks according to the FMEA analysis, especially if they are required to be operated by clinicians or patients. Low risk devices may benefit from demonstrating patient or clinician acceptability, but we do not consider this necessary for first-in-human studies in most cases.

System perspective studies are recommended for devices with high risk. In these cases, potentially high risk first-in-human studies can only be ethically justified if there is a reasonable prospect that the device will ultimately be adopted by the community. High risk devices that do not satisfy an unmet need, or are not economically viable, may therefore not be appropriate for a first-in-human study.

These recommendations (Fig. 3) are based on the guiding ethical principles of the IDEAL collaboration (Box 2), the most important of which is ensuring, as far as possible, that no harm is caused in the stage 1 first-in-human studies.

Box 2 **Guiding Ethical Principles of the IDEAL Collaboration**

Avoiding harm by communicating about any errors or incorrect ideas which were tried and abandoned

Avoiding harm by conducting studies in all fields of interest which seem relevant to the introduction of the specific intervention under discussion

Increasing autonomy by supplying information to the patient

Demonstrating beneficence by testing the reliability and technical effectiveness of techniques and instruments

Studies should of course have a robust design appropriate to the study question and follow relevant reporting guidelines. To ensure maximal general benefit and avoid duplication, studies should be published and subject to peer review. Collation of reports related to a particular device's stage 0 assessment in a centralized resource would allow transparent assessment.^14^ This transparency should not interfere with intellectual property protection and, in fact, should run in parallel to this process. There are useful frameworks to guide developers on what aspects of intellectual property to consider at each stage of the development process.^14,15^

To best illustrate our recommendation and the underlying principles of the IDEAL collaboration, we provide 3 examples to illustrate the process of how the stage 0 recommendations can be tailored to specific devices to optimize the translation to clinical practice (Table 4).

**TABLE 4:** Examples to Illustrate the Process of How the Stage 0 Recommendations Can Be Tailored to Specific Devices to Optimize the Translation to Clinical Practice

(A) Eco-friendly Surgical Drapes		
Device	Nonsurgical, noninvasive.	
Risk	Negligible risk severity and very low risk frequency (eg, finding defects in the drape before application, requiring the opening of another drape), so low risk category.	
Studies	*System*	Not necessary
	*Patient*	Not necessary
	*Clinician*	Not necessary
	*Device*	Compliance with sterility requirements including packaging and longevity of sterility to the displayed expiry dates, can be demonstrated through adherence to relevant ISO standards.

(B) Handheld Extracorporeal Shockwave Lithotripsy		
Device	Noninvasive and surgical (no contact with patients but deliver energy).	
Risk	Serious risks with occasional occurrence (eg, subcapsular renal hematoma), so intermediate risk category.	
Studies	*System*	Not necessary.
	*Patient*	Because patients are usually awake during lithotripsy patient surveys and focus groups would be required to confirm acceptability.
	*Clinician*	Usability of the device needs assessment, particularly in the context of those familiar with existing devices.
	*Device*	Extensive testing needed to support safety and technical effectiveness, including manufacturing simulations and laboratory bench testing.

(C) Software to Improve Safety of Trajectory Planning for Neurosurgical Biopsies of Deep-seated Brain Lesions		
Device	Software used with medical devices falls under the classification of the associated hardware. Because this software controls brain biopsy needles it would be classified as surgical, and an invasive device in contact with the central nervous system.	
Risk	Occasional risks but of possibly critical severity (eg, intracerebral hematoma), so high risk category.	
Studies	*System*	Software necessity and relevance studies including retrospective evaluations of risks (especially bleeding) of current clinical standard (manual) planning.
	*Patient*	Patient surveys and focus groups to assess acceptability of using computer-aided trajectory planning.
	*Clinician*	Focus on the training required to utilize the new software confidently, perhaps via simulated biopsies in phantom patients, demonstrating how the software and user interface could be optimized to minimize the learning curve. Consider how long it takes compared to current clinical standards and, if longer, whether it is worth the potential incremental benefit in terms of safety and/or efficacy.
	*Device*	In view of the risk, extensive evaluations of planned versus actual trajectories in simulations or animal studies could be compared to the planned and actual trajectories in retrospective cases not using the software.

## CONCLUSIONS

These IDEAL-D recommendations for preclinical (stage 0) evaluation of medical devices represent a proportionate and pragmatic approach that balances the de-risking of first-in-human translational studies against the benefits of rapid translation of new devices into clinical practice. They suggest that preclinical evaluation should be tailored to the device classification (Table 1) and to risk stratification of its potential failure modes (Table 2). The recommendations (Fig. 3) are derived from the ethical principles of the IDEAL collaboration (Box 2) and propose a systematic and proportionate approach to selecting appropriate topics for study and study designs from an enormous range of possibilities. We hope that their use will reduce research waste and associated costs and delays, whilst ensuring more appropriate and useful evaluation and less risks of harm during initial clinical studies.

The recommendations have been developed by expert consensus and are not, therefore, evidence based. They, therefore, need to be subjected to empirical testing, and modifications made where proposals prove inappropriate or impractical in case studies. The impact of these recommendations on safety and efficacy aspects of stage 1 studies and the rates of transition from stage 0 to stage 1 should be evaluated.

An important issue, not addressed in these recommendations, is the initial determination of when a device is considered new, and therefore warrants full preclinical assessment within IDEAL-D stage 0. In many cases, it may be unclear whether an alteration in a device's design or use represents a significant change. Future work by the IDEAL collaboration will seek to develop specific recommendations to help innovators make this judgement. In the meantime, we would suggest a group including representatives of innovators, clinicians, scientists, and patients be asked to reach a consensus-based decision based on the ethical principles of the IDEAL collaboration.
